# Radical Prostatectomy in Kidney Transplant Recipients—A Multicenter Experience

**DOI:** 10.1016/j.euros.2024.07.111

**Published:** 2024-07-29

**Authors:** Jacob Schmidt, Abdulbaki Yakac, Robert Peters, Frank Friedersdorff, Karoline Kernig, Anna Kienel, Franziska I. Winterhagen, Friedrich Köpp, Susan Foller, Francesca DiQuilio, Karl Weigand, Luka Flegar, Philipp Reimold, Michael Stöckle, Juliane Putz, Philip Zeuschner

**Affiliations:** a Department of Urology, Charité – Universitätsmedizin Berlin, Corporate Member of Freie Universität Berlin and Humboldt-Universität zu Berlin, Berlin, Germany; b Department of Urology, University Hospital Carl Gustav Carus, Dresden, Germany; c Department of Urology, University of Rostock, Rostock, Germany; d Department of Urology and Pediatric Urology, University Hospital Erlangen, Erlangen, Germany; e Department of Urology, University Hospital Bonn, Rheinische Friedrich-Wilhelms-Universität Bonn, Bonn, Germany; f Department of Urology, Jena University Hospital, Jena, Germany; g Department of Urology, Martin Luther University Halle-Wittenberg, Halle (Saale), Germany; h Department of Urology, Philipps-University Marburg, Marburg, Germany; i Department of Urology and Pediatric Urology, Saarland University, Homburg/Saar, Germany

**Keywords:** Kidney transplantation, Prostate cancer, Prostatectomy, Robot-assisted surgery, Open surgery

## Abstract

**Background and objective:**

Kidney transplant recipients (KTRs) have an increased risk of developing genitourinary cancers, including prostate cancer (PCa), which is expected to become more prevalent due to an aging KTR population. Thus, knowledge of surgical outcomes, including treatment of PCa, within this unique cohort is required.

**Methods:**

Data of 62 KTRs undergoing radical prostatectomy (RP) between 2006 and 2023 at nine urologic transplant centers were analyzed. Complications were assessed using the Clavien-Dindo classification. Perioperative outcomes were evaluated, and a follow-up was conducted. Overall survival (OS), biochemical recurrence–free survival (BRFS), and death-censored graft survival were determined via the Kaplan-Meier method and log-rank testing.

**Key findings and limitations:**

Overall, 50 open radical retropubic RPs and 12 robot-assisted RPs (RARPs) were included. The intraoperative blood loss was lower after RARP, but operative time was longer. Of the patients, 50% experienced no postoperative complication, and grade ≥3 complications were observed in 14.5%. There was no graft loss related to RP. A histopathologic analysis revealed pN1 in 8.1% and positive surgical margins in 25.8% of the cases. At a median follow-up of 48.5 mo, the median OS was 128 (95% confidence interval [CI] 71.2–184.8) mo, BRFS was 106 (95% CI 55.8; 156.2) mo, and graft survival was 127 (95% CI 66.7–187.3) mo. Limitations include the retrospective design, and variations between groups and centers.

**Conclusions and clinical implications:**

Our findings support RP as a feasible and safe treatment option for localized PCa in KTRs with acceptable oncologic outcome. Special care is required in screening and awareness for the risk of understaging.

**Patient summary:**

This study analyzed the safety and effectiveness of two prostate cancer surgery methods—open and robot-assisted surgery—in the special group of kidney transplant recipients. Both surgical methods were performed safely with acceptable oncologic outcomes; however, sample size was too small to draw definite conclusions between the two operative methods.

## Take Home Message

Open and robot-assisted radical prostatectomies are feasible and safe for prostate cancer treatment in kidney transplant recipients, with acceptable oncologic outcomes and minimal impact on graft function using specialized urological expertise in well-selected and curatively intended patients.

## 1 Introduction

Kidney transplantation (KT) is considered the therapeutic gold standard for end-stage renal disease (ESRD), and significantly improves the quality of life and long-term outcomes for recipients compared with dialysis [1]. In kidney transplant recipients (KTRs), genitourinary cancers account for the majority of all noncutaneous cancers [2]. Prostate cancer (PCa) is one of its most common types at an incidence rate of 0.72–3.1% [1], [3], [4], [5]. Over the next few decades, the number of older KTRs is expected to increase due to a shift in demographics. Advances in technology and medicine lead to an increase in life expectancy of about 20 yr for these recipients [6]. Accordingly, the incidence of PCa in this subgroup will presumably rise, increasing the need for precise treatment options and therapy strategies concordantly [4].

However, the presence of a KT before PCa therapy poses a clinical challenge due to the complexities associated with prior dialysis, immunosuppression, pelvic tissue adhesions, and the location of the graft in the iliac fossa, with potential risks of direct and indirect injuries [6]. In localized PCa, radiotherapy (RT) and radical prostatectomy (RP), including open radical retropubic RP (ORRP), endoscopic extraperitoneal RP, laparoscopic RP, and robot-assisted RP (RARP), are effective curative treatment options [1], [6], [7], [8]. Minimally invasive surgical approaches, such as RARP, have attracted attention in recent decades due to their advantages in terms of postoperative recovery and good functional and oncologic outcomes [1], [9]. However, safety as well as clinical and oncologic outcomes of RP for the subgroup of KTRs should be explored in more detail as literature is lacking large series [3], [4], [7], [10], [11], [12], [13], [14]. In a recent retrospective comparison of a KTR with a non-KTR cohort, Marra et al [15] found a significant increase in postoperative complications at a comparable oncologic outcome.

This multicentric retrospective analysis aims to investigate the perioperative and oncologic outcomes of RP, including ORRP and RARP, as a curative therapy of localized PCa after KT, conducted at nine urologic transplant centers.

## 2 Patients and methods

### 2.1 Cohort

We retrospectively included adult patients who underwent RP after KT at nine German transplant centers between 2006 and 2023. We collected relevant patient data from the medical records, including demographics, laboratory parameters, and clinical and pathologic data including a recent follow-up. Biochemical recurrence was defined as a prostate-specific antigen (PSA) level of ≥0.2 ng/ml confirmed by two independent postoperative tests. The surgical techniques for ORRP and RARP were performed as described in the literature [4], [16], [17], [18], [19]. Pelvic lymphadenectomy (PLND) was conducted upon surgeon’s decision. Postoperative complications were assessed according to the Clavien-Dindo classification (CDC) within 30 d [20]. This study was conducted in accordance with the World Medical Association Declaration of Helsinki and was approved by the ethics committee of Charité – Universitätsmedizin Berlin on 12.12.2022 (approval number: EA1/252/22).

### 2.2 Statistical analysis

A statistical analysis was performed using IBM SPSS (Armonk, NY, USA) Statistics 29. Mann-Whitney *U* tests were used for an analysis of continuously coded variables, and chi-square test was used for multiple nominal variables. Overall survival (OS), biochemical recurrence–free survival (BRFS), and death-censored graft survival (GS) were determined using the Kaplan-Meier method and log-rank testing. We defined *p* < 0.05 to indicate statistical significance. Four patients with dialysis dependency and graft failure before RP were excluded from the analysis of the creatinine levels and GS.

## 3 Results

### 3.1 Patient characteristics

Overall, 62 patients were included, of whom 50 (80.6%) underwent ORRP and 12 (19.4%) RARP. The median age at RP was 63.5 (range 32–77) yr and the median body mass index was 25.6 (range 19.1–33) kg/m2. Various forms of glomerulonephritis were the most common ESRD-causing disease with 62.9% (Supplementary Table 1). The median number of antigen mismatches was 3 (range 0–6), and 51.6% of the grafts were located in the left iliac fossa. The median prostate volume was 31 (range 10–85) ml at a preoperative PSA level of 6.5 (range 0–73.2) ng/ml. The median number of tumor-positive biopsies was 4 (range 1–11), and the most common Gleason scores were 7a in 34.5% and Gleason 6 in 27.6%. As depicted in Table 1, cT1c was most prevalent in 30 (48.4%) and cT2a in nine (14.5%) cases. Low- and intermediate-risk tumors according to D’Amico were present in 21 (34.4%) patients each; 19 cases (31.1%) were classified as having a high risk. There was no clinical evidence of distant metastases in any case. Between ORRP and RARP, the patient characteristics did not differ significantly.

**Table 1:** Demographic and preoperative characteristics of patients who underwent radical prostatectomy after kidney transplantation^a^

Characteristics	Overall (*n* = 62)	ORRP (*n* = 50)	RARP (*n* = 12)	*p* value
Age at transplantation (yr)	55.5 (26–76)	55.5 (26–76)	56.5 (35–70)	0.76
Age at radical prostatectomy (yr)	63.5 (32–77)	63.5 (32–77)	64.5 45–77)	0.35
BMI at RP (kg/m^2^)	25.6 (19.1–33)	26.4 (19.1–33)	25.5 (22.4–30)	0.06
Follow-up after NTX (mo)	125 (5–388)	125 (5–388)	125 (55–326)	0.62
Follow-up after RP (mo)	48.5 (0–191)	51 (0–191)	27 (4–118)	0.44
Location of renal transplant				0.90
Left iliac fossa	32 (51.6)	26 (52)	6 (50)	
Right iliac fossa	30 (48.4)	24 (48)	6 (50)	
Number of mismatches	3 (0–6)	3 (0–6)	3.5 (0–6)	0.82
Functioning graft at RP	58 (93.5)	11 (91.7)	47 (94)	0.77
Time interval from transplantation to prostatectomy (mo)	70.5 (4–309)	68.5 (4–274)	76.5 (34–309)	0.36
Preoperative CT scan	20 (33.3)	14 (29.2)	6 (50)	0.17
Preoperative MRI	25 (40.3)	21 (42)	4 (33.3)	0.74
Prostate volume (ml)	31 (10–85)	30 (10–85)	36.5 (15–81)	0.59
Preoperative PSA level (ng/ml)	6.5 (0–73.2)	6.15 (0–73.2)	8.39 (4.87–70.68)	0.19
Number of tumor-positive biopsies	4 (1–11)	4 (1–11)	4.5 (1–8)	0.58
Number of performed biopsies	12 (6–26)	12 (7–26)	12 (6–16)	0.29
Incidental prostate cancer	4 (6.6)	4 (8.2)	0	0.31
Gleason score in biopsy				0.79
6	16 (27.6)	14 (30.4)	2 (16.7)	
7a	20 (34.5)	14 (40.4)	6 (50)	
7b	6 (10.3)	5 (10.9)	1 (8.33)	
8	8 (13.8)	6 (13)	2 (16.7)	
9	6 (10.3)	5 (10.9)	1 8.3)	
10	2 (3.4)	2 (4.3)	0	
Clinical T stage				0.81
cTx	6 (9.7)	5 (10)	1 (8.3)	
cT1a	5 (8.1)	5 (10)	0	
cT1b	1 (1.6)	1 (2)	0	
cT1c	30 (48.4)	21 (42)	9 (75)	
cT2a	9 (14.5)	8 (16)	1 (8.3)	
cT2b	5 (8.1)	4 (8)	1 (8.3)	
cT2c	3 (4.8)	3 (6)	0	
cT3a	2 (3.2)	2 (4)	0	
cT4	1 (1.6)	1 (2)	0	
Clinical positive lymph nodes	3 (6.1)	2 (5.1)	1 (10)	0.75
D'Amico score				0.73
Low	21 (34.4)	16 (32.7)	5 (41.7)	
Intermediate	21 (34.4)	18 (36.7)	3 (25)	
High	19 (31.1)	15 (30.6)	4 (33.3)	

BMI = body mass index; CT = computed tomography; MRI = magnetic resonance imaging; PSA = prostate-specific antigen; ORRP = open retropubic radical prostatectomy; RARP = robot-assisted radical prostatectomy; RP = radical prostatectomy;

^a^ Values are shown as median (range) or *n* (%).

* *p* <0.05 in Mann-Whitney *U* or chi-square test.

### 3.2 Perioperative outcomes

PLND was performed in 43 (69.4%) cases, contralaterally to the graft in 38 (61.3%) and bilaterally in five (8.1%) cases (Table 2). A nerve-sparing approach was performed partially in 16 (26.2%) and completely in 12 (19.7%) cases, while no nerve sparing was conducted in 44 cases (54.1%) . As shown in Table 2, the median operative time was 144 (range 85–236) min. In RARP, the median operative time was 43 min longer, but not statistically significant (176 vs 133 min, *p* = 0.06). The median estimated intraoperative blood loss was 400 (range 100–2000) ml. Blood loss appeared to be higher in ORRP (600 ml, range 100–2000 ml) than in RARP (200 ml, range 100–1500 ml), but showed no statistical significance (*p* = 0.06). Intraoperative complications occurred in six (9.8%) cases. In one case of RARP, conversion to open surgery as well as ureteral stent insertion had to be performed due to an injury of the graft ureter. Severe bleeding with intraoperative blood loss of 1000–1500 ml was reported in three cases, and intraoperative blood transfusion was performed in six (10%) patients, with only one RARP case. In one ORRP case, an acute cardiac event occurred during the operation.

**Table 2:** Surgical and perioperative outcomes ^a^

Characteristics	Overall (*n* = 62)	ORRP (*n* = 50)	RARP (*n* = 12)	*p* value
Lymphadenectomy				0.40
No	19 (30.6)	16 (32)	3 (25)	
Unilateral	38 (61.3)	29 (58)	9 (75)	
Bilateral	5 (8.1)	5 (10)	0	
Number of lymph nodes removed	5 (0–16)	5.5 (0–15)	4 (0–16)	0.62
Nerve sparing				0.30
No	44 (54.1)	27 (55.1)	6 (50)	
Partial	16 (26.2)	11 (22.4)	5 (41.7)	
Complete	12 (19.7)	11 (22.4)	1 (8.3)	
Operative time (min)	144 (85–236)	133 (85–236)	176 (104–230)	0.06
Estimated blood loss (ml)	400 (100–2000)	600 (100–2000)	200 (100–1500)	0.06
Intraoperative complication	6 (9.8)	5 (10.2)	1 (8.3)	0.85
Hemoglobin level (mg/dl)				
Preoperative	13.1 (9.9–16.7)	13.1 (10.2–16.7)	13.1 (9.9–16.3)	0.52
Postoperative day 1	9.6 (6.8–13.4)	9.6 (6.8–13.4)	9.9 (7.5–13.2)	0.88
Postoperative day 3	9.5 (6.8–14.4)	9.5 (6.7–14.4)	9.2 (8.0–12.8)	0.64
Clavien-Dindo (30 d)				0.01 *
No complication	31 (50)	21 (42)	10 (83.3)	
1	3 (4.8)	3 (6)	0	
2	19 (30.9)	19 (38)	0	
3a	3 (4.8)	1 (2)	2 (16.7)	
3b	4 (6.5)	4 (8)	0	
4	2 (3.2)	2 (4)	0	
Creatinine level (mg/dl) ^b^				
Preoperative	1.73 (0.96–3.89)	1.80 (0.96–3.89)	1.72 (1.18–2.76)	0.94
Postoperative day 1	2.06 (0.95–4.50)	2.14 (0.95–4.50)	1.74 (1.45–2.84)	0.67
Postoperative day 3	1.94 (0.85–5.23)	1.99 (0.85–5.22)	1.64 (1.24–2.68)	0.56
Postoperative day 5	1,79 (0.92–5.07)	1.98 (0.92–5.07)	1.74 (1.24–3.11)	0.99
1 mo after surgery	1.76 (0.83–4.40)	1.76 (0.93–4.40)	1.76 (1.20–3.22)	0.85
Hospital stay (d)	8 (4–163)	8 (4–163)	8 (7–20)	0.49

ORRP = open retropubic radical prostatectomy; RARP = robot-assisted radical prostatectomy.

^a^ Values are shown as median (range) or *n* (%).

^b^ Four patients with dialysis dependency were excluded.

* *p* < 0.05 in Mann-Whitney *U* or chi-square test.

The postoperative hemoglobin levels decreased from 13.1 (range 9.9–16.7) to 9.6 (range 6.8–14.4) mg/dl on the 1st postoperative day (POD) and to 9.5 (range 6.8–14.4) mg/dl on the 3rd POD, with no significant differences between ORRP and RARP. Of the patients, 50% had a complication-free course (Table 2). CDC grade 1 and 2 complications were observed in 35.7% of patients, including urogenital infections in six, acute kidney failures in five, conservative treatment of lymphoceles in five, postoperative allogenic blood transfusions in five, and anastomosis insufficiencies in three cases (Supplementary Table 2). CDC grade 3a and 3b complications were observed in 11.3%, with surgical or interventional drainage of a lymphocele in three, wound revision in three, and surgical revision for postoperative bleeding in two cases. There were two cases of myocardial infarction following RP (CDC grade 4). The number and severity of postoperative complications were higher after ORRP than after RARP (*p* = 0.01).

No patient received dialysis during the postoperative course. After a slight increase of creatinine levels on POD 1, it decreased hereafter and did not differ significantly between ORRP and RARP (Table 2).

### 3.3 Oncologic outcome and GS

A histopathologic examination of the RP specimens revealed a majority of pT2c (50%) tumors, followed by pT3a (24.2%) and pT3b (17.7%), as shown in Table 3. Tumor-positive lymph nodes were present in five (8.1%) cases, while no PLND was performed in 19 (30.6%) cases. Sixteen (25.8%) patients had positive surgical margins, with 13 (81.8%) cases having ≥pT3 (50% of ≥pT3 tumors) and three (18.8%) cases having ≤pT2 (8.3% of ≤pT2 tumors) tumors. Lymphovascular invasion was present in six (10%) cases and vascular invasion in one (1.7%) case. The histopathologic Gleason score was predominantly 7a in 25 (40.7%) cases, followed by 7b in 12 (20.3%) cases. There were no significant differences between ORRP and RARP.

**Table 3:** Histopathologic and oncologic outcomes

Characteristics	Overall (*n* = 62)	ORRP (*n* = 50)	RARP (*n* = 12)	*p* value
pT stage				0.49
pT0	2 (3.2)	2 (4)	0	
pT2a	1 (1.6)	1 (2)	0	
pT2b	2 (3.2)	2 (4)	0	
pT2c	31 (50)	25 (50)	6 (50)	
pT3a	15 (24.2)	11 (22)	4 (33.3)	
pT3b	11 (17.7)	9 (18)	2 (16.7)	
pN stage				0.46
pN0	38 (61.3)	31 (62)	7 (58.3)	
pN1	5 (8.1)	3 (6)	2 (16.7)	
pNx	19 (30.6)	16 (32)	3 (25)	
pL stage				0.91
L0	54 (90)	44 (89.8)	10 (90.9)	
L1	6 (10)	5 (10.2)	1 (9.1)	
pV stage				0.63
V0	59 (98.3)	48 (98)	11 (100)	
V1	1 (1.7)	1 (2)	0	
Surgical margins				0.94
R0	46 (74.2)	37 (74)	9 (75)	
R1	16 (25.8)	13 (26)	3 (25)	
≤pT2	3 (18.8)	3 (23.1)	0	
≥pT3	13 (81.8)	10 (76.9)	3 (100)	
Postoperative Gleason score				0.32
6	5 (8.9)	4 (9.1)	1 (8.3)	
7a	24 (42.9)	16 (36.4)	8 (66.7)	
7b	14 (25)	12 (27.3)	2 (16.7)	
8	5 (8.9)	4 (9.1)	1 (8.3)	
9	8 (14.3)	8 (18.2)	0	
PSA level 4–8 wk after surgery (ng/ml)	0.01 (0–23.1)	0.01 (0–23.1)	0.01 (0–3.46)	0.96
Biochemical recurrence	15 (26.8)	11 (24.4)	4 (36.4)	0.42
Biochemical recurrence–free survival (mo)	35 (0–106)	26.5 (0–106)	51 (6–67)	0.39

PSA = prostate-specific antigen; ORRP = open retropubic radical prostatectomy; RARP = robot-assisted radical prostatectomy.

^a^ Values are shown as median (range) or *n* (%).

* *p* < 0.05 in Mann-Whitney *U* or chi-square test.

**Fig. 1:**
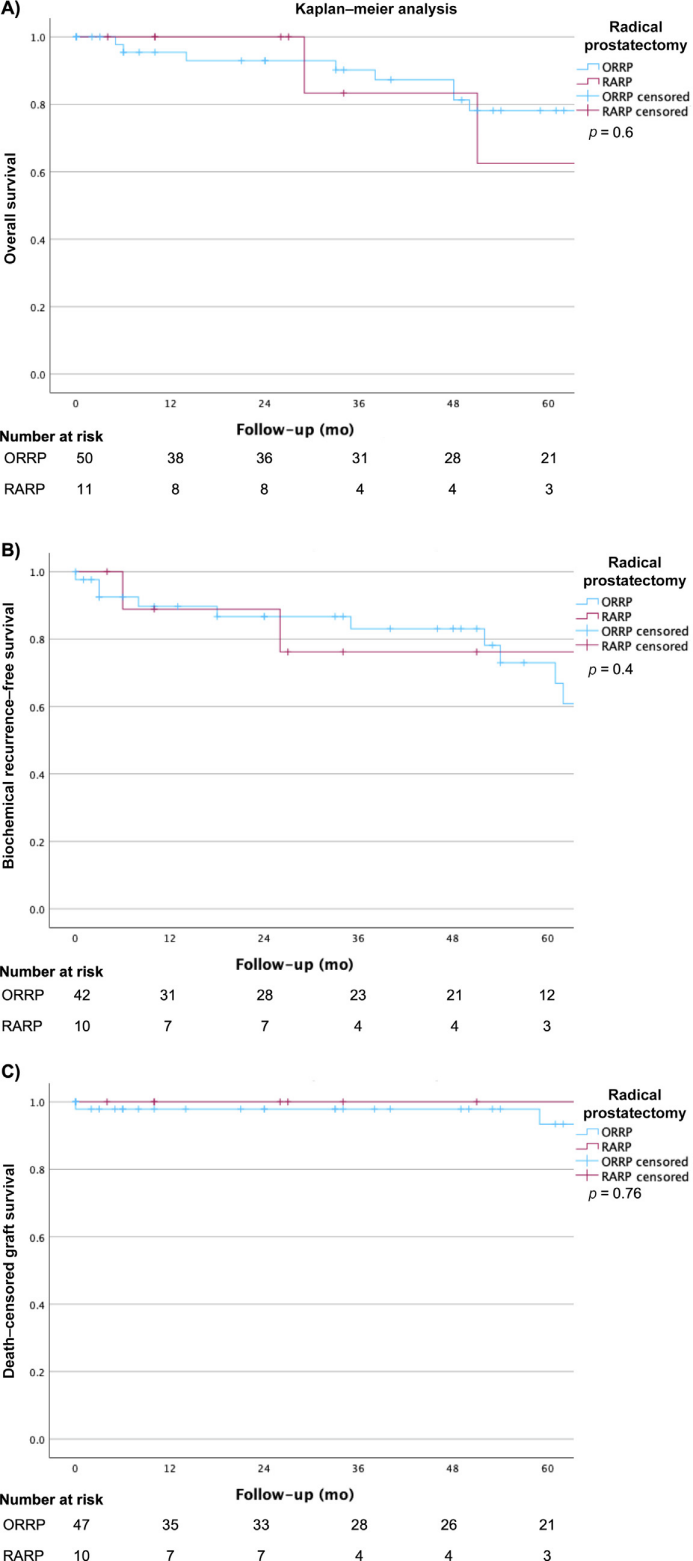
Kaplan-Meier analysis revealed no significant differences in log-rank testing for (A) 5-yr overall survival, (B) biochemical recurrence–free survival, and (C) death-censored graft survival after open retropubic radical prostatectomy (ORRP, red) versus robot-assisted radical prostatectomy (RARP, blue) in kidney transplant recipients.

The median follow-up period after RP was 48.5 (range 0–191) mo after RP and 125 (range 5–388) mo after KT. Overall, 15 (24.6%) patients died of other causes than PCa or RP. The median OS after RP was 128 mo (95% confidence interval [CI] 71.2–184.8), with 128 mo after ORRP and 91 mo after RARP (*p* = 0.4; Fig. 1). The 5-yr OS was 78%, with 80% after ORRP and 64% after RARP (Table 4).

**Table 4:** Overall survival, biochemical recurrence–free survival, and death-censored graft survival

	Overall (*n* = 62)	ORRP (*n* = 50)	RARP (*n* = 12)	*p* value
Overall survival (median)	128 (CI 71.2–184.8)	128	91 (CI 29.4–152.6)	0.60
1 yr	94%	93%	100%	
5 yr	78%	80%	63%	
Biochemical recurrence–free survival (median)	106 (CI 55.8; 156.2)	106 (95% CI 29.3; 183)	67 (33.3; 101)	0.395
1 yr	89%	89%	89%	
5 yr	74%	74%	74%	
Death-censored graft survival (median)	127 (CI 66.7–187.3)	127 (CI 66.5–187.5)	NR	0.758
1 yr	98%	98%	100%	
5 yr	94%	94%	100%	

CI = confidence interval; NR = not reported; ORRP = open retropubic radical prostatectomy; RARP = robot-assisted radical prostatectomy.

^a^ Values are shown as median (range) or %.

* *p* < 0.05 in log-rank test.

The median PSA 4–8 wk after RP was 0.01 (range 0–23.1) ng/ml. During follow-up, a biochemical recurrence occurred in 15 (26.8%) patients at a median of 35 (range 0–106) mo after RP. The median BRFS was 106 (95% CI 55.8–156.2) mo and the 5-yr BRFS was 75% (Table 4). It did not differ between ORRP and RARP (*p* = 0.4), as depicted in Figure 1.

Graft losses due to nonoperative reasons occurred in 16 (26.2%) patients. Cardiorenal causes and chronic graft failure as reasons for graft loss were observed in five (8.1%) cases each. A post–cardiac surgery organ failure, recurrent glomerulonephritis, and post-transplant lymphoproliferative disorder each contributed to one (1.6%) case of graft loss. The cause of graft loss was unknown in three (4.8%) cases. As shown in Table 4, the median death-censored GS after RP was 127 (95% CI 66.7–187.3) mo overall; it was 127 mo (95% CI 66.5–187.5) after ORRP and on average 84 mo after RARP (median not reached, *p* = 0.76).

## 4 Discussion

In the future decades, the incidence of PCa in KTRs is predicted to increase due to demographic changes and an increase in life expectancy after KT [6], [21]. Although RP is considered the gold standard for localized PCa in KTRs, a paucity of available data regarding perioperative and oncologic outcomes in this distinct cohort is eminent [11], [13], [15], [22], [23].

Overall, our findings confirm the feasibility and safety of RP for KTRs with low intraoperative complication rates despite the presence of a graft, previous surgery, and potential adhesions. The data revealed a longer operative time for RARP than for ORRP. Nevertheless, intraoperative blood loss was higher in ORRP. These findings are consistent with those of Basiri et al [24], who compared ORRP and RARP in the non-KTR setting in their systematic review. However, there were no significant differences in postoperative hemoglobin levels between both groups. Furthermore, postoperative complication rates, including the administration of blood transfusions and CDC grade ≥3 complications (14.5%), are consistent with the complication rates reported in the literature [3], [4], [7], [8], [10], [11], [12], [15]. Of note, compared with non-KTR cohorts with CDC grade ≥3 complications of 1–8%, higher complication rates have been demonstrated in the KTR setting, including the present analysis [15], [24]. Nevertheless, it should be considered that the majority of postoperative complications following ORRP were mild (CDC grade 1 or 2). The impact of RP on graft function, as indicated by the transient increase in creatinine levels, emphasizes the need for vigilant postoperative monitoring. The median death-censored GS of 127 mo after RP confirms the safety of RP for the allograft, as no graft loss was attributed directly to RP. Therefore, our results underscore the importance of a meticulous surgical technique, careful preoperative planning, and interdisciplinary postoperative follow-ups to minimize the risk to the graft. Chronic graft failure was identified as one of the most common reasons for graft loss in our cohort. In this context, several perioperative factors need to be considered for their potential impact on long-term GS. Blood transfusions can lead to alloimmunization, a risk factor for chronic rejection, and may compromise GS [25]. However, this has been described only for early blood transfusions after KT, while data are missing on the administration of blood transfusions late in the clinical course, as performed in our cohort with a median time interval between KT and RP of 70.5 mo [25]. In addition, other medical conditions, including cardiac events (3.2% in our cohort), acute renal failure (8.1%), or infections (9.7%), represent other known risk factors for chronic graft failure, as described by Mayrdorfer et al [26]. This is concordant with cardiorenal causes being one of the main reasons for graft loss in our cohort and highlights the vulnerability of this patient population to hemodynamic instability and reduced renal perfusion, which may negatively impact long-term GS. In our retrospective analysis, however, it is not possible to identify a causal relationship between perioperative conditions and implications for long-term GS.

Infections and renal failure were treated directly, and creatinine levels reached the preoperative level at least 1 mo after surgery. Of note, the mentioned complications were relatively rare in our analysis. Most likely, intra- and postoperative complications in our cohort did not have a negative impact on the postoperative mid- and long-term graft function.

In general, PCa is detected at approximately 62.3 yr in KTRs, which is much earlier than in the general population at 70 yr. Moreover, the time from KT to PCa diagnosis is described to be >54 mo [6], [8]. Concordantly, the median age at RP in our cohort of 63.5 yr and the median time from KT to RP of 70.5 mo are within the reported ranges. However, there are differences to other KTR and non-KTR cohorts in the D’Amico risk stratification. With 31.3% high-risk tumors, the present cohort was more frequent than in the comparison of a non-KTR with a KTR cohort by Marra et al [15], with only 17% and 18% high-risk tumors, respectively. Therefore, only definitive treatment was indicated in the majority of our cohort, and a large number of patients had preoperative computed tomography (33.3%) or magnetic resonance (40.3%) imaging.

Lymphoceles were present in eight (12.9%) cases as the most common postoperative complication. In this context, it is particularly important to consider whether or not to perform a PLND. It should be considered for KTRs with a given oncologic risk profile—here, taking into account that the majority of patients had a high or an intermediate risk. In our cohort, the majority (five out of eight) of patients with lymphoceles did not require an intervention or a revision. Consistently with existing series, PLND was conducted only on one side contralateral to the graft in most cases in our cohort [3], [4], [7], [10], [11], [12], [13]. Typically, PLND is not performed on the graft side due to limited space and difficult access.

Furthermore, in this particular patient cohort of KTRs, one should be aware of possible complications, such as an injury of the ureter. The insertion of a ureteral stent for better localization of the graft ureter should be considered in these special cases.

Histopathology revealed 41.9% pT3 tumors—a significant proportion of PCa with extraprostatic extension. This represents a substantial upstaging compared with the preoperative staging of 31.1% high-risk tumors according to D’Amico and cT3 in merely 3.2%. Our results are consistent with Beyer et al’s [13] findings, demonstrating ≥pT3a in 50% of their cohort, but differ from most other series reporting lower pT stages, including that of Marra et al [15] with ≥pT3a in only 29% of their KTR cohort [7], [11], [23]. Although immunosuppression was not previously believed to increase the risk of PCa, our results indicate that careful screening should be performed in KTRs. The risk should not be underestimated clinically, and a nerve-sparing approach should be weighed carefully [27]. With 8.3% positive resection margins in ≤pT2 and 50% in ≥pT3 tumors, our cohort is in the range described for non-KTRs, which is reported to be 5–30% for organ-confined PCa and 17–65% for locally advanced PCa [28]. Despite the possibility of active surveillance in 34.4% cases with low-risk tumors according to D’Amico, this approach should be weighed up carefully in a case-by-case decision as we showed an increased rate of pathologic upstaging and in the cohort of KTRs.

Biochemical recurrence was present in 26.8% of patients with median BRFS of 106 mo and 5-yr BRFS of 74%, which is in the range of recurrence rates reported in the literature for KTR and non-KTR cohorts [23], [24]. Furthermore, the 5-yr OS of our cohort was 78%, which is comparable with other KTR-RP cohorts, as reported by Hevia et al [23], but lower than in the non-KTR setting (95%) [29]. However, no PCa-associated cause of death was reported. Accordingly, it should be emphasized that KTRs remain a patient population with an increased risk profile and numerous comorbidities. Therefore, we recommend treatment in high-volume referral centers with comprehensive multidisciplinary care including specialized urologic expertise to lower the risk for the patient and graft.

Moreover, one has to note that besides RP, other treatment options such as external beam RT, brachytherapy, focal therapy, or active surveillance may also have favorable results in (KT) patients with localized PCa [14]. Each treatment modality has its own advantages and disadvantages, and does not appear to be suitable for every patient. When RT is considered, it should be noted that there is a risk of ureteral stricture and graft damage due to the proximity of the radiation field to the ureter of the graft and salvage RP after RT is associated with significant risks, which may be increased by the presence of a KT [14]. In addition, precise postoperative PSA monitoring and recurrence detection after RP are advantages over RT. One further advantage of RP, particularly in KTRs, again is the urologic expertise in both KT and PCa. Therefore, anatomical and clinical challenges presented by these complex patients can be assessed and managed effectively. As most studies in this field lack long follow-up times, more multicentric prospective studies will be needed to further sharpen the indications for localized PCa therapy in KT patients.

Our analysis has several limitations. The retrospective design, relatively small sample size, and the performance of surgery at different centers with variations in perioperative management should be considered when interpreting the results. Furthermore, differences in group sizes with a majority of ORRP and differences in the follow-up period were present, which limits the comparability of both surgical approaches. Moreover, there is a potential selection bias regarding the individual surgical approaches in each center.

## 5 Conclusions

This multicenter, retrospective analysis emphasizes the safe option of RP in KTRs with localized PCa for curative intended therapy. Our results highlight that RP can be performed with manageable risks, with no significant impact on graft function, and acceptable oncologic control. We emphasize that the KTR cohort requires special care in screening and that there is an eminent risk of pathologic upstaging. Our findings support the need for further research, including larger prospective studies comparing the open and robotic approaches.

  ***Author contributions*:** Jacob Schmidt had full access to all the data in the study and takes responsibility for the integrity of the data and the accuracy of the data analysis.

  *Study concept and design*: Schmidt.

*Acquisition of data*: Schmidt, Yakac, Kernig, Kienel, Winterhagen, Köpp, Foller, DiQuilio, Flegar, Reimold, Zeuschner.

*Analysis and interpretation of data*: Schmidt, Putz, Yakac, Zeuschner.

*Drafting of the manuscript*: Schmidt.

*Critical revision of the manuscript for important intellectual content*: Schmidt, Zeuschner, Putz.

*Statistical analysis*: Schmidt, Yakac, Zeuschner.

*Obtaining funding*: None.

*Administrative, technical, or material support*: Peters, Friedersdorff, Weigand, Stöckle.

*Supervision*: Putz, Zeuschner.

*Other*: None.

  ***Financial disclosures:*** Jacob Schmidt certifies that all conflicts of interest, including specific financial interests and relationships and affiliations relevant to the subject matter or materials discussed in the manuscript (eg, employment/affiliation, grants or funding, consultancies, honoraria, stock ownership or options, expert testimony, royalties, or patents filed, received, or pending), are the following: None.

  ***Funding/Support and role of the sponsor*:** None.

  ***Ethics statement*:** The data presented in this analysis were conducted ethically in accordance with the World Medical Association Declaration of Helsinki. The study was approved by the ethics committee of Charité – Universitätsmedizin Berlin on December 12, 2022 (approval number: EA1/252/22) and supported in all participating centers according to the local conditions for cooperation projects.

## References

[1] Hernández-Gaytán C.A., Rodríguez-Covarrubias F., Castillejos-Molina R.A. (2021). Urological cancers and kidney transplantation: a literature review. Curr Urol Rep.

[2] Putz J., Kestel V., Herout R. (2024). Urogenital tumors following kidney transplantation—monocentric analysis of incidences and overview of urological preventive measures. Urologie.

[3] Moreno Sierra J., Ciappara Paniagua M., Galante Romo M.I. (2016). Robot assisted radical prostatectomy in kidney transplant recipients. Our clinical experience and a systematic review. Urol Int.

[4] Marra G., Agnello M., Giordano A. (2022). Robotic radical prostatectomy for prostate cancer in renal transplant recipients: results from a multicenter series. Eur Urol.

[5] Siegel R.L., Miller K.D., Jemal A. (2020). Cancer statistics, 2020. CA Cancer J Clin.

[6] Marra G., Dalmasso E., Agnello M. (2018). Prostate cancer treatment in renal transplant recipients: a systematic review. BJU Int.

[7] Léonard G., Pradère B., Monléon L. (2020). Oncological and postoperative outcomes of robot-assisted laparoscopic radical prostatectomy in renal transplant recipients: a multicenter and comparative study. Transplant Proc.

[8] Sherer B.A., Warrior K., Godlewski K. (2017). Prostate cancer in renal transplant recipients. Int Braz J Urol.

[9] Marks L.B., Yorke E.D., Jackson A. (2010). Use of normal tissue complication probability models in the clinic. Int J Radiat Oncol Biol Phys.

[10] Zeng J., Christiansen A., Pooli A., Qiu F., Lagrange C.A. (2018). Safety and clinical outcomes of robot-assisted radical prostatectomy in kidney transplant patients: a systematic review. J Endourol.

[11] Felber M., Drouin S.J., Grande P. (2020). Morbidity, perioperative outcomes and complications of robot-assisted radical prostatectomy in kidney transplant patients: a French multicentre study. Urol Oncol Semin Orig Investig.

[12] Shahait M., Al Majali F., Dobbs R.W. (2021). Oncological and functional outcomes of robot-assisted radical prostatectomy in kidney transplant recipients. J Soc Laparoendosc Surg.

[13] Beyer B., Mandel P., Michl U. (2016). Oncological, functional and perioperative outcomes in transplant patients after radical prostatectomy. World J Urol.

[14] Dat A., Wei G., Knight S., Ranasinghe W. (2023). The role of localised prostate cancer treatment in renal transplant patients: a systematic review. BJUI Compass.

[15] Marra G, Tappero S, Barletta F, et al. Radical prostatectomy for nonmetastatic prostate cancer in renal transplant recipients : outcomes for a large contemporary cohort and a matched comparison to patients without a transplant. Eur Urol Focus. In press. 10.1016/j.euf.2024.02.008. 38453584

[16] Coughlin G.D., Yaxley J.W., Chambers S.K. (2018). Robot-assisted laparoscopic prostatectomy versus open radical retropubic prostatectomy: 24-month outcomes from a randomised controlled study. Lancet Oncol.

[17] Magheli A., Gonzalgo M.L., Su L.-M. (2011). Impact of surgical technique (open vs laparoscopic vs robotic- assisted) on pathological and biochemical outcomes following radical prostatectomy: an analysis using propensity score matching. BJU Int.

[18] Busch J., Gonzalgo M.L., Leva N. (2015). Matched comparison of robot-assisted, laparoscopic and open radical prostatectomy regarding pathologic and oncologic outcomes in obese patients. World J Urol.

[19] Altaylouni T., Gebert P., Elezkurtaj S. (2023). Robot-assisted laparoscopic prostatectomy experience and pathological quality: are they always linked?. J Endourol.

[20] Dindo D., Demartines N., Clavien P.A. (2004). Classification of surgical complications: a new proposal with evaluation in a cohort of 6336 patients and results of a survey. Ann Surg.

[21] Sampaio M.S., Cho Y.W., Qazi Y., Bunnapradist S., Hutchinson I.V., Shah T. (2012). Posttransplant malignancies in solid organ adult recipients: an analysis of the U.S. national transplant database. Transplantation.

[22] Heidenreich A., Pfister D., Thissen A., Piper C., Porres D. (2014). Radical retropubic and perineal prostatectomy for clinically localised prostate cancer in renal transplant recipients. Arab J Urol.

[23] Hevia V., Boissier R., Rodríguez-Faba Ó. (2018). Management of localised prostate cancer in kidney transplant patients: a systematic review from the EAU guidelines on renal transplantation panel. Eur Urol Focus.

[24] Basiri A., de la Rosette J.J., Tabatabaei S., Woo H.H., Laguna M.P., Shemshaki H. (2018). Comparison of retropubic, laparoscopic and robotic radical prostatectomy: who is the winner?. World J Urol.

[25] Kang Z.Y., Ma S., Liu W., Liu C. (2023). Effect of blood transfusion post kidney transplantation on de novo human leukocytes antigen donor-specific antibody development and clinical outcomes in kidney transplant recipients: a systematic review and meta-analysis. Transpl Immunol.

[26] Mayrdorfer M., Liefeldt L., Wu K. (2021). Exploring the complexity of death-censored kidney allograft failure. J Am Soc Nephrol.

[27] Al-Adra D.P., Hammel L., Roberts J. (2021). Pretransplant solid organ malignancy and organ transplant candidacy: a consensus expert opinion statement. Am J Transplant.

[28] Koskas Y., Lannes F., Branger N. (2019). Extent of positive surgical margins following radical prostatectomy: Impact on biochemical recurrence with long-term follow-up. BMC Urol.

[29] Stensland K.D., Caram M.E.V., Herr D.J. (2024). National long-term survival estimates after radical prostatectomy for prostate cancer. Urology.

