# Supplementary material for: Radical Prostatectomy in Kidney Transplant Recipients—A Multicenter Experience
Source: Eur Urol Open Sci. 2024 Jul 29;67:45–53. doi: 10.1016/j.euros.2024.07.111 (PMC11339048; doi:10.1016/j.euros.2024.07.111)
Supplement: Supplementary Data 1 [file mmc1.docx]

## Supplementary Table 1. Underlying causes of End-stage renal disease.

| **ESRD causing disease** | **Overall (n=62)** | **ORRP (n=50)** | **RARP (n=12)** |
| --- | --- | --- | --- |
| Unknown | 15 (24.2%) | 14 (28%) | 1 (8.3%) |
| Glomerulonephritis | 39 (62.9%) | 30 (60%) | 9 (75%) |
| Athrophic kidneys | 1 (1.6%) | 1 (2%) | 0 |
| Hypertensive nephropathy | 3 (4.8%) | 1 (2%) | 2 (16.7%) |
| ADPKD | 4 (6.5%) | 4 (8%) | 0 |

**Abbrevations:** ESRD: End Stage Renal Disease; ADPKD: Autosomal dominant polycystic kidney disease

## Supplementary Table 2. Postoperative complications within the first 30 postoperative days; values are shown as number (percentage of the group); *p-value <0.05 in Mann-Whitney U- or Chi-square test.

| **Postoperative complication** | **Overall (n=62)** | **ORRP (n=50)** | **RARP (n=12)** | **p-value** |
| --- | --- | --- | --- | --- |
| Acute kidney failure | 5 (8.1%) | 5 (10%) | 0 | 0.25 |
| Urogenital infection | 6 (9.7%) | 6 (12%) | 0 | 0.21 |
| Lymphocele | 8 (12.9%) | 6 (12%) | 2 (16.7%) | 0.67 |
| Anastomosis insufficiency | 3 (4.8%) | 3 (6%) | 0 | 0.38 |
| Seroma of the abdominal wall | 1 (1.6%) | 1 (2%) | 0 | 0.62 |
| Myocardial infarction | 2 (3.2%) | 2 (4%) | 0 | 0.48 |
| Postoperative blood transfusion | 5 (8.3%) | 5 (10%) | 0 | 0.53 |
| Postoperative bleeding | 2 (3.2%) | 2 (4%) | 0 | 0.48 |
| Wound healing disorder | 2 (3.2%) | 2 (4%) | 0 | 0.48 |
